# Staphylococcal meningitis therapy with linezolid in a young infant: efficacy, CSF levels and side effects

**DOI:** 10.1186/s13052-020-00854-z

**Published:** 2020-06-29

**Authors:** Cinzia Auriti, Fiammetta Piersigilli, Iliana Bersani, Sara Cairoli, Paolina Giuseppina Amante, Daniela Longo, Bianca Maria Goffredo

**Affiliations:** 1 Department of Neonatology, Neonatal Intensive Care Unit, Bambino Gesù Children’s Hospital, IRCCS, Piazza S. Onofrio 4, 00165 Rome, Italy; 2 Department of Pediatric Medicine, Laboratory of Metabolic Biochemistry Unit, Bambino Gesù Children’s Hospital, Bambino Gesù Children’s Hospital, IRCCS, Rome, Italy; 3 Department of Neurosciences, Neurosurgery Unit, Bambino Gesù Children’s Hospital, Rome, Italy; 4 Department of Imaging, Bambino Gesù Childrens’ Hospital, IRCCS, Rome, Italy

**Keywords:** Linezolid, Staphylococcal meningitis, QTc interval, Neonate, Side effect

## Abstract

**Background:**

Linezolid is a synthetic antibiotic which is active against most Gram-positive bacteria, especially on *Staphylococcus aureus*. Its administration can be required when the infection is due to staphylococcus strains, which are resistant to vancomycin. Although mostly well tolerated, some mild to moderate side effects have been reported.

**Case presentation:**

This case report describes an infant with multiloculated hydrocephalus, staphylococcal meningitis and prolonged linezolid therapy, in which we observed the association between linezolid administration and a lengthened QTc interval at the electrocardiogram (ECG). To rule out toxic levels during the therapy, plasma and cerebro-spinal fluid concentrations of linezolid were measured and reported.

**Conclusions:**

Although generally well tolerated in neonates and infants, linezolid prolonged administration seems be able to cause QTc interval prolongation. Therefore, its administration in such patients should be limited to cases of bacterial resistance to other antibiotics. In addition to well-known close monitoring of the platelet level, we suggest serial ECG controls before and during linezolid administration. In the case we report, linezolid plasma concentrations resulted within the therapeutic range during therapy, while cerebrospinal fluid (CSF) concentrations appeared lower than those considered effective.

## Background

Linezolid belongs to a new generation group of synthetic antibiotics, the oxazolidinones, which is active against gram-positive microorganisms, including methicillin-resistant staphylococci, staphylococci with low susceptibility to vancomycin, some gram-negative anaerobic species, and some mycobacterial species (*Mycobacterium tuberculosis* and *Mycobacterium avium complex)*. Linezolid binds to the 23S site of the 50S ribosomal sub-unit, blocks the formation of the 70S ribosomal complex thereby inhibiting protein synthesis. Cross-resistance with other antibiotics seems to be only exceptional. Linezolid has excellent tissue penetration and oral and intravenous administration provide almost equivalent drug availability.

Linezolid administration is currently used in adults when the infection is due to staphylococcus strains resistant to vancomycin, as the drug has been approved by the Food and Drug Administration since 2002. Contrarywise, the clinical experience with linezolid in the pediatric population is still limited [1–4]. There are age-related characteristics in the pharmacokinetic parameters of linezolid. Its half-life in adults and older children is about 4–6 h, suggesting that it can be administered twice a day. Children younger than 12 years have a smaller Area Under the Curve (AUC), a faster clearance and a shorter elimination half-life than adults. Neonates have clearance values that increase markedly during the first week of life, to values 2- to 3-fold in excess of those observed in older children and adults [5–7]. Therefore, a shorter dosing interval is required for neonates to achieve a linezolid exposure similar to that observed in adults receiving 600 mg twice daily and to reach a rate of pathogen eradication similar to that of vancomycin. It has been therefore proposed that children younger than 12 years should receive linezolid 10 mg/kg or more every 8 h [6, 7] only to treat infections due to bacteria resistant to other, best studied drugs.

Although mostly well tolerated, some mild to moderate side effects have however been reported.

In infants and children adverse events, including abnormal kidney function and oral/skin candidiasis, were less frequent with linezolid administration than with vancomycin, although reversible thrombocytopenia after 2 weeks of therapy occurred more frequently with linezolid than the other antibiotic [8].

In adults it has been suggested that bradycardia could represent a further side effect of linezolid, but this has not been confirmed by numerically adequate studies [9].

This case report describes an infant with staphylococcal meningitis and multi-loculated hydrocephalus, requiring a prolonged linezolid therapy, in which we observed the association between linezolid administration and a lengthened QTc interval at the 12 lead ECG.

Plasma and cerebrospinal fluid levels of linezolid were monitored, to rule out toxic levels of the drug.

## Case presentation

A 1.600 g male neonate was delivered by urgent cesarean section at 30 weeks’ gestation. On the third day of life (DOL) he developed a bilateral, grade II-III intraventricular hemorrhage (IVH) associated with enlargement of the lateral, III, and IV ventricles. On the 20th DOL the neonate was referred to our Neonatal Intensive Care Unit to undergo neurosurgery due to a progressive worsening hydrocephalus. He immediately underwent external ventricular drain (EVD), and at 1 month of life a ventriculoperitoneal shunt (VPS) was placed. The VPS was replaced subsequently for several times. It had to be externalized again for the onset of *Escherichia coli* meningitis, requiring 3 weeks of intravenous meropemen and amikacin therapy. The repeated meningeal infections led to a multiloculated hydrocephalus, requiring the diversion of cerebrospinal fluid (CSF) through neuro-endoscopic intra-cystic septostomy and the implantation of three Rickham reservoir devices, in the frontal sites and one in posterior cranial fossa that flowed into the peritoneum.

**Fig. 1:**
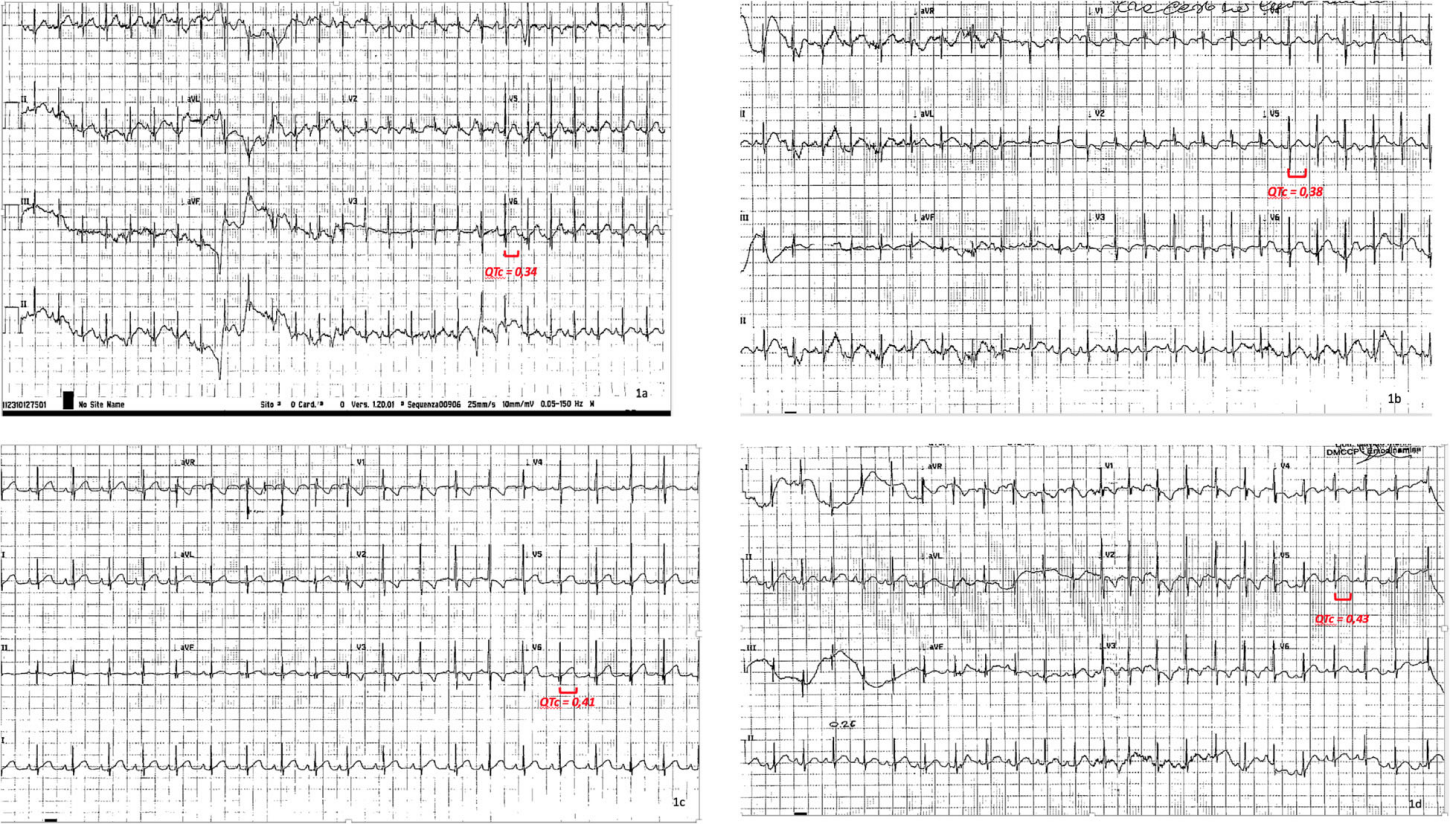
shows the patient’s electrocardiograms before therapy (**a**), at the beginning of linezolid administration (**b**), the progressive prolongation of the QTc tract during (**c**) and at the end of therapy (**d**)

At 8 months of life the infant developed meningitis caused by a *Staphylococcus epidermidis* strain susceptible to vancomycin (MIC 2 μg/ml), to linezolid (MIC 1 μg/ml) and resistant to teicoplanin (MIC 16 μg/ml)*.* Vancomycin therapy (15 mg/Kg, four times in a day) was immediately started but, due to poor response (CSF cultures were persistently positive), after 20 days, intravenous linezolid was added at the dosage of 10 mg/Kg three times in a day, administered in 1 h [5–7, 9]. All the 12 lead ECGs performed up to this time point and during the first days after the start of linezolid had always shown sinus rhythm with QTc interval within the normal range (340 milliseconds) (Fig. 1a and b). About 20 days later a lengthened QTc interval (430 milliseconds) was detected on the ECG (Fig. 1c and d) and confirmed by the examinations carried out in the following days. Suspecting a linezolid-related side effect and because the persistent presence of *Staphylococcus epidermidis* in the CSF cultures, after 24 days of therapy, linezolid was replaced by intravenous rifampicin (20 mg/kg/die) in association with intrathecal vancomycin, with resolution of the meningitis. With the discontinuation of linezolid, QTc interval values returned to the normal range (350–390 milliseconds).

To assess the patient’s proper linezolid exposure and rule out toxic levels, we measured plasma and CSF drug concentrations throughout therapy, by a High-Performance Liquid Chromatography (HPLC). The target to achieve a therapeutic effect was a concentration higher than MIC _90_ of the bacteria considered sensitive (2–4 μg/ml; range of C _max_ described for age: 6.8–36.7 μg/ml) [6, 7]. Linezolid plasma levels resulted within the therapeutic range, while in CSF drug levels appeared to be lower than those considered effective. CSF/plasma concentration ratios were low (Table 1) [5, 6, 10].

**Table 1:** Plasma and CSF levels of linezolid at different time points

Linezolid concentration (μg/ml)^a^			
Time points	Plasma	CSF	CSF/Plasma ratio^b^
1 h before the infusion	7.93	5.57	0.70
1 h after	48.44	4.91	0.10
6 h after	11.25	4.88	0.43

^a^Effective range in infants: C_max_ 6.8–36.7 μg/ml (ref. [6])

^b^CSF/Plasma ratio describing a good penetration into CSF: 0.7–0.8+/− 0.3 (ref. [9])

The neonate was discharged at 1 year of life with severe neurological impairment, under anticonvulsive therapy. Currently he is following our follow up program, receiving periodical pediatric, neurosurgical and cardiologic visits. He did not show any cardiac side effects attributable to linezolid therapy.

## Discussion and conclusions

### Discussion

Linezolid is a weak reversible monoamine oxidase inhibitor and has the potential to interact with adrenergic and serotoninergic agents. When administered with concomitant sympathomimetic agents it can cause an increase in blood pressure and induce heart palpitations. This is the reason why in 2009, the US Food and Drug Administration Agency approved an updated safety labeling for linezolid, pointing out the risks of the simultaneous administration of monoamine oxidase inhibitors, serotonergic agents, and drugs that might increase blood pressure [11].

Most common side effects of Linezolid include gastrointestinal disorders, headache, rash, liver dysfunction, optic and peripheral neuropathy, thrombocytopenia, anemia, neutropenia, lactic acidosis. Those side effects usually occur after prolonged administration and disappear after linezolid discontinuation [3, 4]. To our knowledge, this is the first report describing an association between linezolid administration and a transitory ECG QTc interval prolongation in a preterm born infant. Tartarone described bradycardia in an adult patient after 48 h of therapy with linezolid, but QTc was normal [9] A single study performed in adult patients assessed the potential link between linezolid and QTc prolongation, without finding apparent relationships [12]. Rubinstein performed a Phase III study and found that QTc intervals measured before and after the administration of linezolid vs placebo were similar [13, 14]. However, there are currently no available data investigating the potential development of arrhythmias or cardiac complication among neonates or infants treated with linezolid.

In our patient, the lengthened QTc interval appeared after a prolonged administration of linezolid and disappeared after drug discontinuation. This side effect was reported as adverse event to the Italian Agency for Drug Monitoring (AIFA).

Furthermore, in our patient the prolonged treatment with linezolid did not resolve the meningitis from *Staphylococcus epidermidis*. Although plasma levels of the drug were in the range of effectiveness, ratios between CSF and plasma concentrations were lower than the optimal value. Our patient had a multiloculated hydrocephalus and it is easy to hypothesize that the CSF circulation could be compromised by the particular anatomical feature, hindering the uniform linezolid distribution into the liquor cysts. Linezolid CSF levels assessed at different time points from the administration seem to confirm this altered drug distribution (Table 1).

## Conclusions

According to our experience, although generally well tolerated, linezolid prolonged administration seems able to cause a prolongation of the QTc interval in neonates and infants. Therefore, its administration in such patients should be limited to cases of bacterial resistance to other antibiotics. Moreover, in addition to the already known necessity for close monitoring of the platelet level, during linezolid administration we suggest serial ECG controls, before and during the therapy, in particular if the administration lasts more than 2 weeks.

In our patient therapy with linezolid did not resolve the meningitis from *Staphylococcus epidermidis*. Although plasma levels of the drug were in the correct range, ratios between CSF and plasma concentrations were lower than the optimal value. Well-designed studies on linezolid administration to neonates and infants are required to confirm our findings.

## Data Availability

Data supporting the results reported in the article can be found in the intranet of Bambino Gesù Childrens’ Hospital.

## Abbreviations

AIFA: Italian Agency for Drug Monitoring
AUC: Area under the curve
CSF: Cerebro spinal fluid
DOL: Day of life
ECG: Electrocardiogram
EVD: External ventricular drain
HPLC: High-performance liquid chromatography
IVH: Intraventricular hemorrhage
MIC: Minimal inhibitory concentration
QTc: Corrected QT interval of the electrocardiogram
VPS: Ventriculoperitoneal shunt

## References

[1] Mendes RE, Deshpande LM, Costello AJ, Farrell DJ (2012). Molecular epidemiology of Staphylococcus epidermidis clinical isolates from US hospital. Antimicrob Agents Chemother.

[2] Norrby R (2001). Expert Opin Pharmacother.

[3] Stevens DL, Herr D, Lampiris H, Hunt JL, Batts DH, Hafkin B (2002). Linezolid versus vancomycin for the treatment of methicillin-resistant Staphylococcus aureus infections. Clin Infect Dis.

[4] Shibata Y, Yamagishi Y, Mikamo H, Kato H, Nishiyama N, Asai N, Koizumi Y, Matsuura K, Suematsu H, Hagihara MJ. Comparative study on safety of linezolid and vancomycin in the treatment of infants and neonates for gram-positive bacterial infections. Infect Chemother. 2018. 10.1016/j.jiac.2018.04.006. 10.1016/j.jiac.2018.04.006 29807867

[5] Andes D, van Ogtrop ML, Peng J, Craig WA (2002). In vivo pharmacodynamics of a new oxazolidinone (linezolid). Antimicrob Agents Chemother.

[6] Jungbluth GL, Welshman IR, Hopkins NK (2003). Linezolid pharmacokinetics in pediatric patients: an overview. Pediatr Infect Dis J.

[7] Li S-C, Ye Q, Xu H, Zhang L, Wang Y. Population pharmacokinetics and dosing optimization of linezolid in pediatric patients. Antimicrob Agents Chemother. 2019, 2019. 10.1128/AAC.02387-18. 10.1128/AAC.02387-18 PMC6437496 30642929

[8] Deville JG, Adler S, Azimi PH, Jantausch BA, Morfin MR, Beltran S (2003). Linezolid versus vancomycin in the treatment of known or suspected resistant gram-positive infections in neonates. Pediatr Infect Dis J.

[9] Tartarone A, Gallucci G, Iodice G, Romano G, Coccaro M, Vigliotti ML, Mele G, Matera R (2004). Linezolid–induced bradycardia: a case report. Int J Antimicrob Agents.

[10] Dryden MS. Linezolid pharmacokinetics and pharmacodynamics in clinical treatment. J Antimicrob Chemother. 2011, 2011;66(Suppl 4). 10.1093/jac/dkr072. 10.1093/jac/dkr072 21521707

[11] Waknine Y (2008). FDA safety changes: mirena, zyvox, orencia.

[12] Damle B, LaBadie R, Cuozzo C, Alvey C, Chong C, Choo H (2011). Lack of an effect of linezolid on QTc interval prolongation. Antimicrob Agents Chemother.

[13] Rubinstein E, Isturiz R, Standiford HC, Smith LG, Oliphant TH, Cammarata S (2003). Worldwide assessment of linezolid's clinical safety and tolerability: comparator-controlled phase III studies. Antimicrob Agents Chemother.

[14] Vinh DC, Rubinstein E. Linezolid: a review of safety and tolerability. J Infect. 2009, 2009;59(Suppl 1). 10.1016/S0163-4453(09)60009-8. 10.1016/S0163-4453(09)60009-8 19766891

